# Bologna guidelines for diagnosis and management of adhesive small bowel obstruction (ASBO): 2013 update of the evidence-based guidelines from the world society of emergency surgery ASBO working group

**DOI:** 10.1186/1749-7922-8-42

**Published:** 2013-10-10

**Authors:** Salomone Di Saverio, Federico Coccolini, Marica Galati, Nazareno Smerieri, Walter L Biffl, Luca Ansaloni, Gregorio Tugnoli, George C Velmahos, Massimo Sartelli, Cino Bendinelli, Gustavo Pereira Fraga, Michael D Kelly, Frederick A Moore, Vincenzo Mandalà, Stefano Mandalà, Michele Masetti, Elio Jovine, Antonio D Pinna, Andrew B Peitzman, Ari Leppaniemi, Paul H Sugarbaker, Harry Van Goor, Ernest E Moore, Johannes Jeekel, Fausto Catena

**Affiliations:** 1 Emergency and Trauma Surgery Unit, Departments of Emergency and Surgery, Maggiore Hospital Trauma Center, Bologna, Italy; 2 Emergency Surgery Unit, Department of General and Multivisceral Transplant Surgery, S Orsola Malpighi University Hospital, Bologna, Italy; 3 Upper GI Unit, Department of Surgery, Frenchay Hospital, North Bristol, NHS Trust, Bristol, UK; 4 Department of Surgery, Denver Health, University of Colorado Health Sciences Denver, Denver Health Medical Center, 777 Bannock Street, Denver CO 80204, USA; 5 General Surgery I, Ospedali Riuniti di Bergamo, Bergamo, Italy; 6 Department of General and Emergency Surgery, Associated Hospitals “Villa Sofia - Cervello”, Palermo, Italy; 7 Department of Surgery, Massachusetts General Hospital, Harvard Medical School, Boston, MA, USA; 8 Department of Surgery, Macerata Hospital, Via Santa Lucia 2, 62100 Macerata, Italy; 9 Washington Cancer Institute, Washington Hospital Center, Washington, 20010 DC, USA; 10 Department of Surgery, Radboud University Nijmegen Medical Centre, P.O. Box 9101 6500 HB, Nijmegen, The Netherlands; 11 Department of Surgery, University of Florida, Gainesville, FL 32610-0254, USA; 12 Department of Surgery, Erasmus University Medical Center, PO Box 2040 3000 CA, Rotterdam, The Netherlands; 13 Department of Surgery, John Hunter Hospital and University of Newcastle, Locke Bag 1 Hunter Region Maile Centre, Newcastle, NSW 2310, Australia; 14 Department of Emergency and Trauma Surgery, Maggiore Hospital of Parma, Parma, Italy; 15 Emergency Surgery, Department of Abdominal Surgery, Meilahti Hospital, University of Helsinki, Haartmaninkatu 4, 340, Helsinki FIN-00029 HUS, Finland; 16 Division of General Surgery, University of Pittsburgh Physicians, Pittsburgh 15213 PA, USA; 17 Division of Trauma Surgery, University of Campinas, Campinas, SP, Brazil

## Abstract

**Background:**

In 2013 Guidelines on diagnosis and management of ASBO have been revised and updated by the WSES Working Group on ASBO to develop current evidence-based algorithms and focus indications and safety of conservative treatment, timing of surgery and indications for laparoscopy.

**Recommendations:**

In absence of signs of strangulation and history of persistent vomiting or combined CT-scan signs (free fluid, mesenteric edema, small-bowel feces sign, devascularization) patients with partial ASBO can be managed safely with NOM and tube decompression should be attempted. These patients are good candidates for Water-Soluble-Contrast-Medium (WSCM) with both diagnostic and therapeutic purposes. The radiologic appearance of WSCM in the colon within 24 hours from administration predicts resolution. WSCM maybe administered either orally or via NGT both immediately at admission or after failed conservative treatment for 48 hours. The use of WSCM is safe and reduces need for surgery, time to resolution and hospital stay.

NOM, in absence of signs of strangulation or peritonitis, can be prolonged up to 72 hours. After 72 hours of NOM without resolution, surgery is recommended.

Patients treated non-operatively have shorter hospital stay, but higher recurrence rate and shorter time to re-admission, although the risk of new surgically treated episodes of ASBO is unchanged. Risk factors for recurrences are age <40 years and matted adhesions. WSCM does not decrease recurrence rates or recurrences needing surgery.

Open surgery is often used for strangulating ASBO as well as after failed conservative management. In selected patients and with appropriate skills, laparoscopic approach is advisable using open access technique. Access in left upper quadrant or left flank is the safest and only completely obstructing adhesions should be identified and lysed with cold scissors. Laparoscopic adhesiolysis should be attempted preferably if first episode of SBO and/or anticipated single band. A low threshold for open conversion should be maintained.

Peritoneal adhesions should be prevented. Hyaluronic acid-carboxycellulose membrane and icodextrin decrease incidence of adhesions. Icodextrin may reduce the risk of re-obstruction. HA cannot reduce need of surgery.

Adhesions quantification and scoring maybe useful for achieving standardized assessment of adhesions severity and for further research in diagnosis and treatment of ASBO.

## Background of WSES guidelines

Adhesive small bowel obstruction requires appropriate management with a proper diagnostic and therapeutic pathway. Indication and length of Non Operative treatment and appropriate timing for surgery may represent an insidious issue.

Delay in surgical treatment may cause a substantial increase of morbidity and mortality. However repeated laparotomy and adhesiolysis may worsen the process of adhesion formation and their severity. Furthermore the introduction and widespread of laparoscopy has raised the question of selection of appropriate patients with ASBO good candidate for laparoscopic approach. On the other hand, several adjuncts for improving the success rate of NOM and clarifying indications and timing for surgery are currently available, such as hyperosmolar water soluble contrast medium.

No consensus has been reached in diagnosing and managing the patients with ASBO and specific and updated guidelines are lacking.

We carried out an extensive review of the English-language literature and found that there was little high-level evidence in this field, and no systematically described practical manual for the field. Most importantly, there are no standardized diagnostic criteria and therapeutic management guidelines for ASBO, therefore, we would like to establish standards for these items. The Bologna Guidelines include evidence-based medicine and reflect the international consensus obtained through earnest discussions among professionals in the field on 1–3 July, 2010, at the Belmeloro Convention Center, Bologna, Italy.

We aimed to validate and refine the first version of the guidelines, hypothesizing that a model, incorporated in a treatment algorithm, would be predictive, would prevent delayed management of strangulation and would be successfully improved.

**Figure 1:**
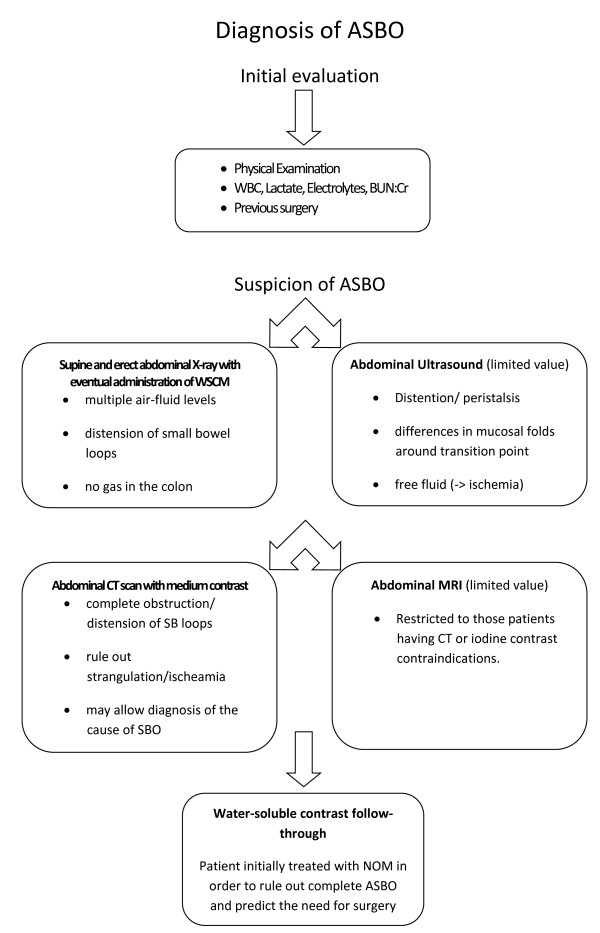
Evidence-based Algorithm for Diagnosis and Assessment of ASBO.

**Figure 2:**
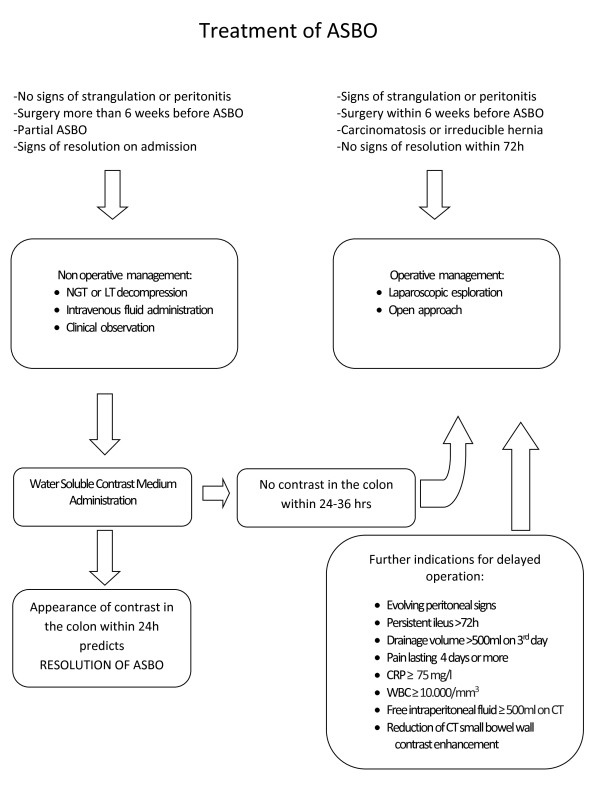
Evidence-based Algorithm for Management and Treatment of ASBO.

Therefore in 2013 the guidelines have been revised and updated by the WSES Working Group on ASBO with the development of diagnosis and treatment evidence-based algorithms (Figure 1, Figure 2).

Furthermore a customary management can help to standardize care throughout a district, a region, or a state satisfying the corporate governance requirements of “clinical efficacy” and “economic efficiency” with the results of improved outcomes and decreased costs.

Improvement of performance is a mainstay of any practice management guideline.

### Notes on the use of the guidelines

The Guidelines are evidence-based, with the grade of recommendation also based on the evidence. The Guidelines present the diagnostic and therapeutic methods for optimal management and prevention of ASBO.

The practice Guidelines promulgated in this work do not represent a standard of practice. They are suggested plans of care, based on best available evidence and the consensus of experts, but they do not exclude other approaches as being within the standard of practice. For example, they should not be used to compel adherence to a given method of medical management, which method should be finally determined after taking account of the conditions at the relevant medical institution (staff levels, experience, equipment, etc.) and the characteristics of the individual patient. However, responsibility for the results of treatment rests with those who are directly engaged therein, and not with the consensus group.

### Definition

Abdominal adhesions, which can begin forming within a few hours after an operation, represent the most common cause of intestinal obstruction being responsible for 60% to 70% of SBO [1,2]. Adhesional postoperative small bowel obstruction is characterized by the presence of abdominal pain, vomiting, distention, and obstipation, in conjunction of confirmatory imaging.

### Risk factors

Patients with ASBO treated nonsurgically have shorter hospital stay, however they have an higher recurrence rate, shorter time to re-admission, although the risk of new surgically treated episodes of ASBO is the same (Level of Evidence 2b).

SBO can be classified according to completeness: Partial vs. Complete (or high grade vs. low grade), according to etiology: Adhesional vs. Non-adhesional, according to timing: Early vs. Late (>30 days after surgery).

Even if Zielinski and Bannon proposed to switch the traditional focus of differentiating SBO to one of predicting failure of NOM with the goal of exploring patients with expected failure as soon as possible [3].

The most important risk factor for adhesive SBO is the type of surgery and extent of peritoneal damage.

The technique of the procedure (open VS laparoscopic) play an important role in the development of adhesion related morbidity. In a retrospective review of 446.331 abdominal operation, Galinos et al. noticed that the incidence was 7.1% in open cholecystectomies vs 0.2% in laparoscopic; 15.6 in open total abdominal hysterectomies vs 0.0% in laparoscopic; 23.9% in open adnexal operations vs 0.0% in laparoscopic and there was no significant difference between open and laparoscopic appendectomies (1.4% vs 1.3%) [4].

In a further recent paper Reshef et al. compared the risk of ASBO in 205 patients who underwent laparoscopic colorectal surgery and 205 who underwent similar open operations, both without any previous history of open surgery. After a mean follow-up of 41 months the authors found that although the rate of admission for ASBO was similar (9% vs 13%, p = 0.3 for the laparoscopic and the open group), the need for operative intervention for ASBO was significantly lower after laparoscopic operations (2% vs 8%, p = 0.006). These data suggest that the lower incidence of adhesions expected after laparoscopic surgery likely translates into long-term benefits in terms of reduced SBO [5].

Other well-known risk factors include surgeries of the colon and rectum (i.e. total colectomy with ileal pouch-anal anastomosis), gynecologic surgeries, age younger than 60 years, previous laparotomy within 5 years, peritonitis, multiple laparotomies, emergency surgery, omental resection, and penetrating abdominal trauma, especially gunshot wounds, a high number of prior episodes of ASBO [1-10].

### Initial evaluation

After an accurate physical examination and the evaluation of WBC, Lactate, Electrolytes, BUN/Creat; first step of diagnostic work up for ASBO is supine and erect plain abdominal X-ray which can show multiple air-fluid levels, distension of small bowel loops and the absence of gas in the colonic section [11].

All patients being evaluated for small bowel obstruction should have plain films (Level of Evidence 2b GoR C).

### Secondary evaluation

CT scan is highly diagnostic in SBO and has a great value in all patients with inconclusive plain films for complete or high grade SBO [12]. However CT-scans should not be routinely performed in the decision-making process except when clinical history, physical examination, and plain film are not conclusive for small bowel obstruction diagnosis [13] (Level of Evidence 2b GoR B).

CT scan often allows to confirm the presence of complete obstruction, to reach the diagnosis of the cause of SBO, it also exclude a non-adhesional pathology and assess the occurrence of strangulation with a sensitivity and specificity higher than 90% and a NPV of nearly 100% [14].

The association of CT scan signs of bowel ischemia should lead a low threshold for surgical intervention (Level of Evidence 2a GoR B).

Ultrasound has a limited value in bowel obstruction or in patients with distended bowel, because the air may obscure the underlying findings. Usual US findings are: distention, peristalsis (differential diagnosis of ileus vs. mechanical SBO), differences in mucosal folds around transition point, free fluid (sign of ischemia) [15].

MRI use should be restricted to those patients having CT or iodine contrast contraindications (Level of Evidence 2c GoR C).

Water-soluble contrast follow-through is valuable in patients undergoing initial non operative conservative management in order to rule out complete ASBO and predict the need for surgery [16] (Level of Evidence 1b GoR A). Water-soluble contrast administration has both diagnostic and therapeutic value [17,18].

This investigation is safer than barium in cases of perforation and peritoneal spread and has possible therapeutic value in the case of adhesive small intestine obstruction [19].

### Conservative treatment and timing for surgery

The management of ASBO is controversial because surgery can induce new adhesions, whereas conservative treatment does not remove the cause of the obstruction [20]. Conservative treatment involves nasogastric intubation, intravenous fluid administration, and clinical observation. Strangulation of the bowel requires immediate surgery, but intestinal ischemia can be difficult to determine clinically.

Potentially, acute care surgery (ACS) model may adversely affect patients who present with SBO because they may be handed over from surgeon to surgeon without definitive care. These patients may not require an operation initially but may require one subsequently because of the development of complications or if the SBO does not resolve with conservative treatment.

In an Australian retrospective study Lien et al. observed that, in the ACS period, there was no significant difference in complication rates or length of hospital stay in those who were not handed over and those who were, both in the pre-ACS and ACS period.

The authors suggested that clinical handover may provide an 'audit-point’ for patient management and opportunity for collaborative input. Moreover, participation of doctors with greater clinical experience may minimize errors in information transfer due to increased acumen in recognizing potential complications [21].

A delay in operation for SBO places patients at higher risk for bowel resection. In a retrospective review Leung and coll find that younger patients (P = 0.001), no previous operation (P < 0.001), and absence of adhesive disease (P < 0.001) were more likely to go to operation. Acquiring a CT scan (P = 0.029) or radiograph (P < 0.001) were factors that increased time to the operating room (OR). In the group with time to OR less than 24 hours, 12% of patients had bowel resection versus time to OR greater than 24 hours, 29% of them required bowel resection [22].

Several issues are raised when managing patients with ASBO.

### Operative management VS Non operative management

Patients without the signs of strangulation or peritonitis or history of persistent vomiting or combination of CT scan signs (free fluid, mesenteric edema, lack of feces signs, devascularized bowel) and partial ASBO can safely undergo non-operative management (LoE 1a GoR A). In these patients tube decompression should be attempted (Level of Evidence 1b GoR A), either with NGT or LT [23].

In conservatively treated patients with ASBO, the drainage volume through the long tube on day 3 (cut-off value; 500 mL) was the indicator for surgery [24].

Also in patients with repeated episodes and many prior laparotomies for adhesions, prolonged conservative treatment (including parenteral nutritional support) may be prudent and often avoid a complex high-risk procedure [25], but the use of supplementary diagnostic tools might be desirable to find the patients who will need early operative treatment [26].

Patients who had surgery within the six weeks before the episode of small bowel obstruction, patients with signs of strangulation or peritonitis (fever, tachycardia and leucocytosis, metabolic acidosis and continuous pain), patients with irreducible hernia and patients who started to have signs of resolution at the time of admission are NOT candidate for conservative treatment +/- WSCA administration (Level of Evidence 1a GoR A) [27,28].

Complete SBO (no evidence of air within the large bowel) and increased serum creatine phosphokinase predicts NOM failure (Level of Evidence 2b GoR C). Free intraperitoneal fluid, mesenteric edema, lack of the “small bowel feces sign” at CT, and history of vomiting, severe abdominal pain (VAS > 4), abdominal guarding, raised WCC and devascularized bowel at CT predict the need for emergent laparotomy at the time of admission (Level of Evidence 2c GoR C).

The appearance of water-soluble contrast in the colon on abdominal X ray within 24 hours of its administration predicts resolution of ASBO (Level of Evidence 1a GoR A). Among patients with ASBO initially managed with a conservative strategy, predicting risk of operation is difficult.

Tachycardia, fever, focal tenderness, increased white blood cell counts, and elevated lactate levels can indicate intestinal ischemia, but these indicators are not very specific [29]. When intestinal ischemia is unlikely, a conservative approach can be followed for 24–48 h.

Zielinski and Bannon in a recent review suggest to combine data from oral contrast meal with their predictive model which identifies patients with mesenteric edema, lack of the small bowel feces signs and obstipation from 12 hours at high risk. The authors recommend urgent exploration for any patient presenting with signs of strangulation or all three of the new model features on admission and without contrast in the colon within 8 hours of administration [3].

Moreover Schraufnagel et al. in n univariate analyses shown that complications, resection, prolonged length of stay and death are more likely in patients admitted for ASBO and operated on the fourth day or later [30].

### Non operative management

There are no advantages with the use of long tube decompression compared with the use of nasogastric tubes (Level of Evidence 1b GoR A) [23,31].

However early tube decompression, either with long or nasogastric tube, may be beneficial (Level of Evidence 2b GoR C) in the initial management of non strangulating ASBO, in adjunct with fluid resuscitation and electrolytes imbalances correction. For challenging cases of ASBO, the long tube should be placed as soon as possible [24] more advisable by endoscopy, rather than by fluoroscopic guide [32].

The use of Gastrografin in ASBO is safe (in terms of morbidity and mortality) and reduces the need for surgery, the time to resolution of obstruction and the hospital stay (Level of Evidence 1a GoR A) [16,19,33-35]. Nevertheless anaphylactoid reaction and lethal aspiration have been described [36].

Gastrografin may be administered on the dosage of 50–150 ml, either orally or via NGT and can be given both at immediately admission or after an attempt of initial traditional conservative treatment of 48 hours (Level of Evidence 1b GoR A).

Regarding the therapeutic value of Gastrografin, some authors affirmed that water-soluble contrast reduces the hospital stay but does not reduce the need for surgery [27,37,38], others has proven that is effective in both reducing the need for surgery and shortening hospital stay, without differences in complications and mortality [28].

As further adjuncts needs to be mentioned that oral therapy with magnesium oxide, L. acidophilus and simethicone may hasten the resolution of conservatively treated partial ASBO and shorten the hospital stay (Level of Evidence 1b GoR A) [39].

To be thorough it has to be mentioned Hyperbaric oxygen (HBO) therapy, that appears to be beneficial in older patients with high anesthesiologic risk (Level of Evidence 2b GoR B). HBO therapy may be an option in the management of patients for whom surgery should be avoided [40].

### Indication for delayed operation

Usually NOM, in absence of signs of strangulation or peritonitis, can be prolonged up to 72 hours of adhesive SBO (Level of Evidence 2b GoR C) [41].

After 3 days without resolution, WSCA study or surgery is recommended (Level of Evidence 2b GoR C) [31].

If ileus persists more than 3 days and the drainage volume on day 3 is > 500 ml, surgery for ASBO is recommended (Level of Evidence 2b GoR C) [24].

With closely monitoring and in the absence of signs suggestive of complications, an observation period even longer than 10 days before proceeding to surgical intervention appears to be safe [42].

However at any time, if onset of fever and leukocytosis greater than 15 000/mm3 (predictors of intestinal complications) are observed, then NOM should be discontinued and surgery is recommended.

The patients non responders to the long-tube and conservative treatment within 72 hours have a considerable risk of recurrent ASBO (Level of Evidence 2b GoR C).

Risk factors for recurrences are age <40 years, matted adhesion (Level of Evidence 1b GoR A) and postoperative surgical complications [43].

Gastrografin use does not affect the recurrences rates or recurrences needing surgery when compared to traditionally conservatively treated patients (Level of Evidence 1b GoR A) [19].

### Surgical treatment: open VS laparoscopic approach

Open surgery is the preferred method for the surgical treatment of strangulating ASBO and after failed conservative management (LOE 2c GOR C).

In highly selected group of patients the laparoscopic can be attempted using an open access technique (LOE 2c GOR C).

The access in the left upper quadrant should be safe (LOE 4 GOR C).

Laparoscopic lysis of adhesions should be attempted preferably in case of first episode of SBO and/or anticipated single band adhesion (i.e. SBO after appendectomy or hysterectomy) (LOE 3b GOR C).

A low threshold for open conversion should be maintained if extensive adhesions are found (LOE 2c GOR C).

Conversion to laparoscopic-assisted adhesiolysis (mini-laparotomy with an incision less than 4 cm long) or laparotomy should be considered in those patients presenting with dense or pelvic adhesion (LOE 3b GOR C).

The extent of adhesiolysis is a matter still under debate. The approaches to adhesiolysis for bowel obstruction among general surgeons in the United Kingdom were established in 1993 [44]. Half of all surgeons divided all adhesions to prevent recurrence of bowel obstruction, whereas the other half limited adhesiolysis to only the adhesions responsible for the obstruction.

The risk of anterior abdominal wall adhesions increases with the number of previous laparotomies although this relationship is not as evident as the relationship between previous laparotomies and adhesiolysis-induced enterotomy [45,46].

Higher age and higher number of previous laparotomies appeared to be predictors of the occurrence of inadvertent enterotomy [46]. Patients with three or more previous laparotomies had a 10-fold increase in enterotomy compared with patients with one or two previous laparotomies strongly suggesting more dense adhesion reformation after each reoperation.

Historically, laparotomy and open adhesiolysis have been the treatment for patients requiring surgery for small bowel obstruction. Unfortunately, this often leads to further formation of intraabdominal adhesions with approximately 10% to 30% of patients requiring another laparotomy for recurrent bowel obstruction [29].

In animal models laparoscopy has been shown to decrease the incidence, extent, and severity of intraabdominal adhesions when compared with open surgery, thus potentially decreasing the recurrence rate for adhesive small bowel obstruction [47].

Tolutope and Scott administered a questionnaire to all the general surgeons registered in the state of Connecticut, trying to know their opinions about the use of laparoscopic lysis of adhesions (LLA) to manage adhesive small bowel obstruction compared with open lysis of adhesions (OLA) in terms of safety, contraindications, and outcomes.

According to their self-reports, 60% of the respondents used LLA in their practice, with 38% of this group using LLA for less than 15% of their adhesive SBO cases. Compared with surgeons out of training more than 15 years, a greater number of surgeons out of training less than 15 years considered LLA to be safer (P = 0.03) and to have better outcomes (P = 0.04) than OLA. More surgeons in academic/teaching hospitals considered LLA to be safe than did surgeons in nonacademic/nonteaching settings (P = 0.04), and more members of the Society of American Gastrointestinal and Endoscopic Surgeons/ Society of Laparoendoscopic Surgeons, considered LLA to be safe than nonmembers (P = 0.001).

These data suggest that recent training and interest or membership in minimally invasive surgery associations influence surgeons’ choice for laparoscopic lysis of adhesions [48].

Laparoscopy seems to have an advantage above laparotomy in terms of adhesion formation to the abdominal wall and to the operative site [49,50], both because of no further scar on anterior parietal peritoneum and because usually the exploration of the ileum is limited to solve the cause of obstruction, extending the dissection until the ligament of Treitz only when the cause of obstruction is not be detected [51].

Laparoscopic adhesiolysis for small bowel obstruction has a number of potential advantages: (1) less postoperative pain, (2) faster return of intestinal function, (3) shorter hospital stay, (4) reduced recovery time, allowing an earlier return to full activity, (5) decreased wound complications, and (6) decreased postoperative adhesion formation [52,53].

These data have been validated in a meta-analysis in which Ming-Zhe Li et al. found that there was no statistically significant difference between open versus laparoscopic adhesiolysis in the number of intraoperative bowel injuries, nor for wound infections, neither with respect to the overall mortality. Conversely there was a statistically significant difference concerning pulmonary complications and a considerable reduction in prolonged ileus in the laparoscopic group compared with the open group. The authors sustain that laparoscopic approach is safer than the open procedure, but in the hands of experienced laparoscopic surgeons in selected patients [54].

Besides Stephanian et al. observed that minimal trauma, short duration of the operation, good cosmetic results and uncomplicated course of postoperative period witness the efficacy of laparoscopic approach [55].

In a consensus conference on laparoscopic adhesiolysis, an italian panel of experts recommended intraoperative selection of patients after esploratory laparoscopy, because this approach allows as many patients as possible to benefit from this mini-invasive procedure. They agree that the only absolute exclusion criteria for laparoscopic adhesiolysis in SBO are those related to pneumoperitoneum (i.e. hemodynamic instability or cardiopulmonary impairment); all other contraindication are relative and shoud be judjed on a case-to-case basis, depending on the laparoscopic skills of the surgeon. Moreover non resolving partial incomplete SBO(after a negative Gastrografin test) and chronic obstructive symptoms are the ideal application for laparoscopic adhesiolysis with rates of conversion as low as 8.7% [56].

However no randomized controlled trial comparing open to laparoscopic adhesiolysis exists up to date, and both the precise indications and specific outcomes of laparoscopic adhesiolysis for adhesive SBO remain poorly understood. The only RCT on laparoscopic adhesiolysis assessed the incidence of chronic abdominal pain after randomization to laparoscopic adhesiolysis or no treatment during diagnostic laparoscopy and it failed to demonstrate any significant differences in terms of pain or discomfort [57].

Although data from a retrospective clinical controlled trial suggest that laparoscopy seems feasible and better in terms of hospital stay and mortality reduction [58].

In a retrospective analyisis Grafen et al. compared the outcomes of laparoscopic management of ASBO to both exploratory laparotomy and secondary conversion to open surgery. 93 patients were divided into successful laparoscopy (71%), secondary conversion (26%) and primary laparotomy (3%). The first group had more simple adhesions, fewer prior operations, lower ASA score, shortest operative time, as was the duration of both intensive care unit and hospital stay; moreover they were younger and had a shorter duration of SBO prior to their operation. Despite that mortality was 6%, regardless of operative technique. The authors, moreover, found that patients who only had prior appendectomy or cholecystectomy could all be managed laparoscopically without need for secondary conversion; on the other hand a prolonged ileus (mean 4.3 days) with progressive abdominal distension and a higher number or more demanding previous operations address to a primary laparotomy. Finally the reasons for converting to open adhesiolysis were: inadequate laparoscopic control due to intestinal distension, extensive adhesions, iatrogenic perforations and resection of necrotic segments [59].

When deciding between an open or laparoscopic approach, the first consideration is that the surgeon be trained and capable of performing advanced laparoscopy.

With regards to patient selection, individuals with an acute small bowel obstruction and peritonitis, free air or gangrenous bowel requiring an emergent operation are best managed with a laparotomy. Patients without peritonitis who do not resolve with nonoperative management should be considered for laparoscopic adhesiolysis. In these cases, it is important to consider the bowel diameter, degree of abdominal distention, and location of the obstruction (ie, proximal or distal). Suter et al. [60] found that a bowel diameter exceeding 4 cm was associated with an increased rate of conversion: 55% versus 32%. Patients with a distal and complete small bowel obstruction have an increased incidence of intraoperative complications and increased risk of conversion. Patients with persistent abdominal distention after nasogastric intubation are also unlikely to be treated successfully with laparoscopy.

The influence of dense adhesions and the number of previous operations on the success of laparoscopic adhesiolysis is controversial. León et al. state that a documented history of severe or extensive dense adhesions is a contraindication to laparoscopy [61]. In contrast, Suter et al. found no correlation between the number and or type of previous surgeries and the chance of a successful laparoscopic surgery [60]. Other factors such as an elevated white blood cell count or a fever have not been demonstrated to correlate with an increased conversion rate. One group of patients who are good candidates for laparoscopic adhesiolysis are those with a nonresolving, partial small bowel obstruction or a recurrent, chronic small bowel obstruction demonstrated on contrast study [61,62].

In an Irish systematic review of over 2000 cases of ASBO, 1284 (64%) were successfully treated with a laparoscopic approach, 6.7% were lap-assisted, and 0.3% were converted to hernia repair; the overall conversion rate to midline laparotomy was 29%. Dense adhesions, bowel resection, unidentified pathology and iatrogenic injury accounted for the majority of conversions. When the etiology was attributed to a single-band adhesion, the success rate was 73.4%. Morbidity and mortality were respectively 14.8% and 1.5%. The inadvertent enterotomy rate was 6.6%. In this perspective laparoscopy seems to be feasible and effective treatment for ASBO with acceptable morbidity [63].

Navez et al. reported that when the cause of obstruction was a single band, laparoscopic adhesiolysis was successful 100% of the time [64].

When other etiologies are found, such as internal hernia, inguinal hernia, neoplasm, inflammatory bowel disease, intussusception, and gallstone ileus, conversion to a minilaparotomy or a formal laparotomy is often required.

Inadvertent enterotomy during reopening of the abdomen or subsequent adhesion dissection is a feared complication of surgery after previous laparotomy. The incidence can be as high as 20% in open surgery and between 1% and 100% in laparoscopy [65].

The incidence of intraoperative enterotomies during laparoscopic adhesiolysis ranges from 3% to 17.6%, with most authors reporting an incidence of about 10% [66,67].

One of the most dreaded complications of surgery is a missed enterotomy. Although a missed enterotomy can occur after laparotomy, the incidence is higher after laparoscopic surgery.

The long-term results regarding recurrence are limited, with most series reporting a mean follow-up between 12 and 24 months.

Feasibility of diagnostic laparoscopy is ranging from 60% to 100% whilst therapeutic effectiveness of the laparoscopic approach is lower (40-88%). Predictive factors for successful laparoscopic adhesiolysis are: number of previous laparotomies ≤2, non-median previous laparotomy, appendectomy as previous surgical treatment causing adherences, unique band adhesion as pathogenetic mechanism of small bowel obstruction, early laparoscopic management within 24 hours from the onset of symptoms, no signs of peritonitis on physical examination, experience of the surgeon [68,69].

Surgical operating time is greater in patients who underwent laparoscopic surgery compared to patients who underwent a laparotomy [70,71].

Postoperative morbidity is lower in patients who underwent laparoscopic adhesiolysis compared to those who underwent the laparotomic approach. Furthermore a greater rate of morbidity is present in patients who underwent laparotomic conversion; whereas mortality is comparable in the two groups (0-4%). Finally the laparoscopic adhesiolysis can avoid laparotomy, which is itself a cause of new adhesions and bowel obstruction, although some authors noticed a greater incidence of recurrent small bowel obstructions in patients who underwent laparoscopy compared to those in which a laparotomy was performed [72,73].

Operative technique has a capital role for a successful laparoscopic treatment [52]. The initial trocar should be placed away (alternative site technique) from the scars in an attempt to avoid adhesions. Some investigators have recommended the use of computed tomography scan or ultrasonography to help determine a safe site for the initial trocar insertion.

The left upper quadrant or the left flank are usually the safest safe place to gain access to the abdominal cavity. Alternatively a 10 mm port can be inserted in the left flank with two additional 5 mm ports in the left upper and lower quadrant (or 10 mm and 5 mm respectively) [74]. Therefore, by triangulating 3 ports aimed at the right lower quadrant, a good exposure and access to the right iliac fossa can be obtained and a technique running the small bowel in a retrograde fashion, starting from the ileocecal valve (decompressed intestine) proximally towards the transition point between collapsed and dilated loops.

The open (Hasson) approach under direct vision is the more prudent. Once safe access is obtained, the next goal is to provide adequate visualization in order to insert the remaining trocars. This often requires some degree of adhesiolysis along the anterior abdominal wall. Numerous techniques are available, including finger dissection through the initial trocar site and using the camera to bluntly dissect the adhesions. Sometimes, gentle retraction on the adhesions will separate the tissue planes. Most often sharp adhesiolysis is required. The use of cautery and ultrasound dissection should be limited or possibly avoided in order to prevent thermal tissue damage and bowel injury [74].

The risk of enterotomy can be reduced if meticulous care is taken in the use of atraumatic graspers only and if the manipulation of friable, distended bowel is minimized by handling the mesentery of the bowel whenever possible [74]. In fact to handle dilated and edematous bowel during adhesiolysis is dangerous and the risk increases with a long lasting obstruction; this is the reason why early operation is advisable as one multicenter study showed: the success rate for early laparoscopic intervention for acute SBO is significantly higher after a shorter duration of symptoms (24 h vs 48 h) [75].

After trocar placement, the initial goal is to expose the collapsed distal bowel [74]. This is facilitated with the use of angled telescopes and maximal tilting/rotating of the surgical table. It may also be necessary to move the laparoscope to different trocars to improve visualization. Only pathologic adhesions should be lysed. Additional adhesiolysis only adds to the operative time and to the risks of surgery without benefit. The area lysed should be thoroughly inspected for possible bleeding and bowel injury.

In conclusion, careful selection criteria for laparoscopy [76] may be: (1) Hemodynamic stability and patient not in shock, (2) absence of peritonitis or severe intra-abdominal sepsis, (3) proximal i.e. SB obstruction, (4) localized distension on radiography, and/or (5) absence of severe abdominal distension, (6) anticipated single band, (7) low or intermediate predicted PAI score in < = 3 abdominal quadrants, and last but not least (8) the experience and laparoscopic skills of the surgeon. A partial obstruction is better first approached with a non-operative challenge with hyperosmolar water soluble contrast medium with both therapeutic and diagnostic purposes. A complete SB obstruction should no longer be considered an exclusion criteria for laparoscopic approach.

The experts panel also agreed, as from the cited studies, that laparoscopic lysis of adhesions should be attempted preferably in case of first episode of SBO and/or anticipated single band adhesion (i.e. SBO after appendectomy or hysterectomy). Previous midline incision is not an absolute exclusion criteria for laparoscopic approach.

A multicenter series of 103 patients from the WSES - Iitalian Working Group on peritoneal adhesions and ASBO management, presented at the 2013 Clinical Congress of American College of Surgeons [77], described a safe and effective surgical technique for laparoscopic approach to ASBO and confirmed that laparoscopy should be attempted preferably in case of first episode of SBO and/or anticipated single band adhesion (i.e. SBO after appendectomy or hysterectomy). A low threshold for open conversion should be maintained if extensive adhesions are found, as often occurs in patients with previous midline laparotomy and multiple surgeries. Previous midline laparotomy incision and multiple previous episodes of ASBO with estimated PAI score of > =2 in more than 3 abdominal regions, were significantly associated in this series with increased risk of conversion and longer operative times.

### Prevention

We do need to prevent ASBO (LOE 2b GoR B).

In view of the incidence of adhesions and recurrence rates of ASBO as well as of the magnitude of the medical problems and financial burden related to adhesions, prevention or reduction of postoperative adhesions in an important priority. Hyaluronic acid-carboxycellulose membrane and icodextrin are able to reduce adhesions (respectively LOE 1a GOR A and LOE 1b GOR A).

Icodextrin may reduce the risk of re-obstruction for ASBO (LOE 1 b GOR A).

Hyaluronic acid-carboxycellulose can not reduce the need of surgery for ASBO (LOE 1a GOR A).

Most of the available literature is based on gynecologic patients. For general surgical patients no recommendations or guidelines exist.

Any prevention strategy should be safe, effective, practical, and cost effective. A combination of prevention strategies might be more effective [78].

In the same review the authors recommend a laparoscopic approach if possible, the use of bioabsorbable barriers, a meticulous hemostasis, avoiding excessive tissue dissection and ischemia and reducing remaining surgical material [78].

In the long term follow up study from Fevang et al. [79] the surgical treatment itself decreased the risk of future admissions for ASBO, even though the risk of new surgically treated ASBO episodes was the same regardless of the method of treatment (surgical vs conservative).

Intraoperative techniques such as avoiding unnecessary peritoneal dissection, avoiding spillage of intestinal contents or gallstones [80], and the use of starch-free gloves [81-83] are basic principles that should be applied to all patients.

In most abdominal procedures the laparoscopic approach is associated with a significantly lower incidence of adhesive SBO or adhesion-related re-admission [79,83].

There is some class I evidence in obstetrics supporting the theory that suturing the peritoneum increases the risk of adhesions [84].

Concerning mechanical barriers no progresses has been made in the last 6 years. The authors remain convinced that the absorbable adhesion barrier Interceed reduces the incidence of adhesion formation following laparoscopy and laparotomy [85-90]. Gore-Tex may be superior to Interceed in preventing adhesion formation but its usefulness is limited by the need for suturing and later removal [91]. There was no evidence of effectiveness of Seprafilm and Fibrin sheet in preventing adhesion formation [92-99].

Chemical/fluid agents have the theoretical advantage of covering more potential sites of adhesion formation than mechanical barriers.

In the newest P.O.P.A. study Catena et al. randomized 91 patients to have 2000 cc of icodextrin 4% and 90 to have the traditional treatment. The authors noted no significant difference in the incidence of small bowel leakage or anastomotic breakdown; operative times, blood losses, incidence of small bowel resections, return of bowel function, LOS, early and late morbility and mortality was comparable. After a mean follow-up of 41.4 months, there have been 2 cases of ASBO recurrence in the icodextrin group and 10 cases in the control group (p < 0.05). Only one patient in the first group was submitted to surgery showing an Adhesion Severity Score = 2, whereas three patients in the latter group were operated, and the ASS was respectively 3,2 and 3. In accordance with this data, the use of icodextrin 4% solution seems to be safe and effective to prevent intra-abdominal adhesion formation and the risk of re-obstruction [100].

Intergel solution (Lifecore Biomedical, Inc, Chaska, MN), which contains .5% ferric hyaluronate, is another product used for adhesion prevention. In preliminary studies it has been shown to reduce the number, severity, and extent of adhesions in peritoneal surgery [101]. However, the use of Intergel in abdominal surgery in which the gastrointestinal tract was opened still led to an unacceptably high rate of postoperative complications [102].

An interesting experimental finding is the reduction of both number and type of adhesions after postoperative stimulation of gastrointestinal motility by a prokinetic agent [103].

Finally merits mention that peritoneal infusion with cold saline has shown to decrease the degree of postoperative intra-abdominal adhesion formation in an animal model [104].

### Adhesions quantification

**Figure 3:**
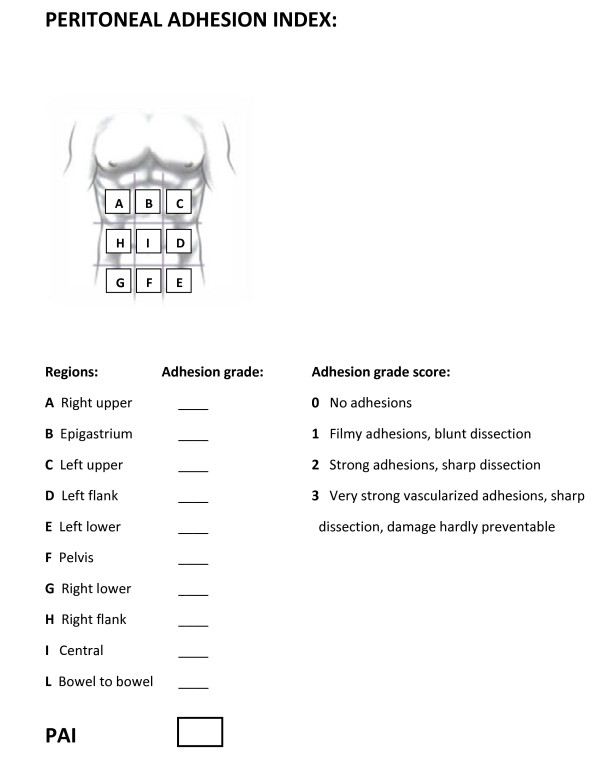
Peritoneal adhesion index: by ascribing to each abdomen area an adhesion related score as indicated, the sum of the scores will result in the PAI.

Among the different adhesions scoring systems which have been proposed mainly by gynecologists, the more complete and easy to use one is the PAI score proposed by Coccolini et al. [105]. In fact, specific attention should be paid to uniformity of measurement. We therefore suggest a regimented classification system for adhesions in an effort to standardize their definition and subsequent analysis. In this way, different surgeons in different treatment centers can more effectively evaluate patients and compare their conditions to past evaluations using a universal classification system (Figure 3). This classification is based on the macroscopic appearance of adhesions and their extent to the different regions of the abdomen. Using specific scoring criteria, clinicians can assign a peritoneal adhesion index (PAI) ranging from 0 to 30, thereby giving a precise description of the intra-abdominal condition [105].

## Conclusions

ASBO is a common disease. Non operative management should be attempted in absence of signs of peritonitis or strangulation. WSCM is safe and has a definite role in diagnosis (for predicting the resolution or need for surgery) and therapy (for reducing the operative rate and shortening time to resolution of symptoms and hospital stay). Open surgery for several surgeons still remains the safest and most effective operative approach, although laparoscopic approach appears to be safe and feasible in the hands of experienced laparoscopic surgeons and in selected patients, because there are less overall complications, prolonged ileus rates and pulmonary complication associated with its use. Prevention with hyaluronic acid-carboxycellulose membrane or icodextrin, has actually gained a capital relevance. Adhesions quantification and scoring is a promising development tool for further research towards diagnosis and management of ASBO and peritoneal adhesions prevention.

## Competing interests

The authors declare that they have no competing interests.

## Authors’ contributions

FC, SDS: conception and design of the study; organised the consensus conference; preparation of the draft; merged the committee preliminary statements with the observations and recommendations from the panel, summarised the discussion on standards of diagnosis and treatment for ASBO SDS, FC, MG, FeCo manuscript writing, drafting and review. FC, SDS, MDK, JJ organised the consensus conference, merged the committee preliminary statements with the observations and recommendations from the panel, critically contributed to the consensus statements. MDK, WLB, LA, VM, HVG, EEM, JJ contributed to critical discussion of the draft. All authors read and approved the final manuscript.
